# Eastchester clapping sign and networks related to spatial attention

**DOI:** 10.1055/s-0042-1758394

**Published:** 2022-12-19

**Authors:** Gustavo José Luvizutto, Gabriel Pereira Braga, Luiz Eduardo Gomes Garcia Betting, Rodrigo Bazan

**Affiliations:** 1 Universidade Federal do Triângulo Mineiro, Departamento de Fisioterapia Aplicada, Uberaba MG, Brazil.; 2 Universidade Federal do Mato Grosso do Sul, Hospital Universitário Maria Aparecida Pedrossian, Campo Grande MS, Brazil.; 3 Universidade Estadual Paulista Júlio de Mesquita Filho, Faculdade de Medicina de Botucatu, Departamento de Neurologia, Psicologia e Psiquiatria, Botucatu SP, Brazil.

**Figure 1:**
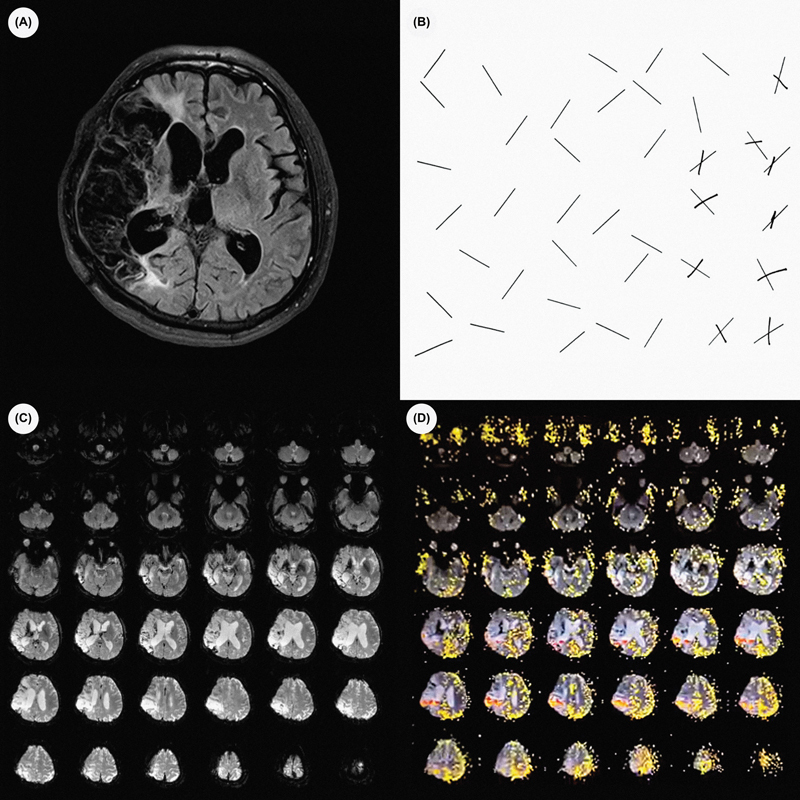
( **A** ) FLAIR MRI scan showing extensive ischemic stroke in the right hemisphere; ( **B** ) line cancellation test (Albert test) indicating presence of unilateral spatial neglect; ( **C** ) BOLD fMRI study at rest showing no activation; ( **D** ) BOLD fMRI study showing bilateral activation of the parietal cortex during sensory stimulation (face-hand test).

A 53-year-old right-handed man developed left hemiparesis (of grade 2 in the Medical Research Council Scale) and right head deviation due to ischemic stroke. When instructed to clap his hands, he brought his right hand to the midline and searched for the other hand ( Video 1 ). Fluid-attenuated inversion recovery (FLAIR) magnetic resonance imaging (MRI) scans, the unilateral spatial neglect (USN) test, and the blood-oxygen-level-dependent (BOLD) functional magnetic resonance imaging (fMRI) study are presented in Figure 1 .

**Video 1** Eastchester clapping sign (ECS-1 = searching in the contralateral hemispace for the other hand). In the video, the neurologist dictates the following command to the patient: “Please clap your hands.”

Link: https://www.arquivosdeneuropsiquiatria.org/wp-content/uploads/2022/04/ANP-2022.0020-video.mov


The Eastchester clapping sign provides evidence of USN phenomena. 1 2 Frequently, patients with USN can ignore problems with the affected limb. This patient presented bilateral activation in the networks related to spatial attention (mainly parietal posterior lobes), and fMRI patterns indicated maladaptive plasticity. 3 4
